# Frequency of undiagnosed hypertension among diabetic patients with micro vascular complications

**DOI:** 10.12669/pjms.41.1.9715

**Published:** 2025-01

**Authors:** Sarah Shoaib Qureshi, Wasim Amer, Maryam Mueez, Mehrin Farok, Syed Khurram Shehzad

**Affiliations:** 1 Sarah Shoaib Qureshi, Department of Medicine, Lahore Medical and Dental College, Lahore, Pakistan; 2 Wasim Amer, Department of Medicine, Lahore Medical and Dental College, Lahore, Pakistan; 3 Maryam Mueez, Department of Medicine, Lahore Medical and Dental College, Lahore, Pakistan; 4 Mehrin Farok, Department of Medicine, Lahore Medical and Dental College, Lahore, Pakistan; 5 Syed Khurram Shehzad, Department of Medicine, Lahore Medical and Dental College, Lahore, Pakistan

**Keywords:** Diabetes, Micro vascular Complications, Hypertension

## Abstract

**Objectives:**

To determine the frequency of undiagnosed hypertension among the diabetic patients with micro vascular complications.

**Method:**

This is a descriptive case series conducted at Department of Medicine, Ghurki Trust Teaching Hospital, in this six month stud which enrolled 213 patients between 18-60 years from March 28, 2021 to September 28, 2021, having diabetes with microvascular complications. These patients were not previously diagnosed as hypertensives. Patient was diagnosed as hypertensive if SBP or DBP was ≥l40 or ≥90 mmHg respectively. The frequency of undiagnosed hypertension was noted and compared across various subgroups of patients.

**Results:**

The mean age of the patients was 49.3±9.7 years. The mean BMI of these patients was 28.3±3.5 Kg/m2 and 67 (31.5%) patients were obese. The mean duration of disease was 8.2±3.9 years. Majority (62.4%) of the patients had diabetic retinopathy while 37.6% patients had diabetic nephropathy. Undiagnosed hypertension was observed in 42 (19.7%) patients with diabetic micro vascular complications. The frequency of undiagnosed hypertension was significantly higher among obese as compared to non-obese patients (28.4% vs. 15.8%; p-value=0.032). There was no statistically significant difference in the frequency of undiagnosed hypertension across various subgroups based on patient’s age (p-value=0.750), gender (p-value=0.902), type of micro vascular complication (p value=0.783) and duration of diabetes (p-value=0.763).

**Conclusion:**

In the present study, a substantial proportion of patients with diabetic micro vascular complications suffered undiagnosed hypertension which is alarming and advocates routine blood pressure monitoring of such patients so that timely identification and anticipated management of underlying hypertension may improve the outcome of such cases in future clinical practice

## INTRODUCTION

Diabetes Mellitus is the pandemic of current century with developing world including Pakistan, being most effected.^1^ Not only the number of people with diabetes is increasing but are being diagnosed at an earlier age. This translates into more people living with diabetes in their productive years, increased lost work days, more health expenditure and morbidity at an earlier age.

Diabetes is associated with a lot of complications and most of the mortality and morbidity in people with diabetes is a consequence of vascular complications.^2^ Hypertension is one of the most common co morbidity in diabetics and multiplies the incidence of complications.^3^ Hypertension not only contributes to the development of macro and micro vascular diabetic complications, but also exacerbate the preexisting diabetic complications.^4^ Coexisting hypertension in people with diabetes is attributed to the risk CV events by 41% and death by 44%, as compared to 9% and 7% of risks in people with diabetes without hypertension.^3^ Prevalence of hypertension in people with diabetes is due to hyperglycemia, dyslipidemia and insulin resistance. These lead to vascular inflammation and increased atherosclerosis.^5^

Hypertension acts as a “silent killer” due to being asymptomatic in most of the patients.^6^ According to UKPDS around 40% of Type-2 diabetes are hypertensive increasing to 60% by the age of 75.^7^ Undiagnosed hypertension leads to development of new or exacerbation of already developed complications. Rodrigues et al. in 2010 conducted a study on the diabetic patients and found that 18.9% of the patients with diabetic retinopathy had undiagnosed hypertension and 16.6% patients with diabetic nephropathy had undiagnosed hypertension.^8^ There are scarce local published data present on this topic. Hence this study was conducted in local population who have developed microvascular complications without previously being diagnosed as hypertensive. The frequency of undiagnosed hypertension among the diabetic patients with retinopathy, neuropathy and nephropathy should be determined. This will allow timely management of these patients helping in reducing the mortality and morbidity.

## METHOD

This descriptive case series was conducted at Department of Medicine, Ghurki Trust Teaching Hospital (GTTH), Lahore.Duration of study was six months from March 28, 2021 to September 28, 2021. Sample size of 213 cases was calculated with 95% confidence level and 5% margin of error while taking expected frequency of masked hypertension among the diabetic patients with diabetic nephropathy to be 16.6%.^8^ Patients were selected by non-probability, consecutive sampling. Patients were considered diabetics according to ADA 2021 criteria.^9^

### Ethical Approval:

It was obtained from Ethical Committee / IRB approval (Reference No: LM&DC/3456 dated March 16, 2021).

Hypertension was diagnosed on two BP readings of systolic blood pressure (SBP) >l40mmHg or diastolic blood pressure (DBP) >90mmHg at two separate occasions eight hours apart and patient not taking antihypertensive medication.

### Inclusion & Exclusion criteria:

Patients having a diagnosis of diabetic retinopathy or diabetic nephropathy were considered as having microvascular complications for this study. Patients of both genders, 18-60 years who had diabetes and micro vascular complications were included. Patients already taking antihypertensive, had renal impairment (serum creatinine >1.2mg/dl) as per history and investigations, patients who had cardiac problem (ejection fraction <45%), had a pervious episode of myocardial infarction or stroke as per clinical record and investigations were excluded

Written informed consent and detailed history was taken from each patient. The diabetic patients who presented in the outpatient department and not previously confirmed as having microvascular complications underwent the fundoscopy and slit lamp examination of the eye. Urine samples were collected at three different occasions eight hours apart in 24 hours and were sent to measure urinary albumin excretion rate. The patients already having or newly diagnosed diabetic retinopathy and / or nephropathy were asked to get their blood pressure checked by a doctor after every eight hours for next 72 hours and keep record of it. Then they were asked to revisit after 72 hours for BP checkup. The presence of undiagnosed hypertension was noted as per operational definition.

### Statistical Analysis:

All the collected data was analyzed through SPSS version 25.0.1. Numerical variables i.e. age, BMI and duration of diabetes have been presented by mean ±SD. 2. Categorical variables i.e. gender, type of micro vascular complication and undiagnosed hypertension has been presented as frequency and percentage. Data has been stratified for age, gender, BMI, duration of diabetes and type of micro vascular complication to address effect modifiers. Post stratification, chi-square test has been applied taking p value ≤0.05 as significant.

## RESULTS

The age of the patients ranged from 18 years to 60 years with a mean of 49.3±9.7 years. Majority (n=153, 71.8%) of the patients were aged 40 years and above. The BMI of these patients ranged from 21.5 Kg/m2 to 34.5 Kg/m2 with a mean of 28.3±3.5 Kg/m2. 67 (31.5%) patients were obese. The duration of disease ranged from 2-15 years with a mean of 8.2±3.9 years. Baseline characteristics of patients in the study are shown in Table-I.

**Table-I:** Baseline Characteristics of Study Sample.

Characteristics	Participant (n=213)
Age (years)	49.3±9.7
>40 years	153 (71.8%)
< 40 years	60 (28.2%)
***Gender***	
Male	125 (58.7%)
Female	88 (41.3%)
BMI (Kg/m2)	28.3±3.5
Non-Obese	146 (68.5%)
Obese	67 (31.5%)
Duration of Diabetes (years)	8.2±3.9
2-8 years	116 (54.5%)
9-15 years	97 (45.5%)
***Micro vascular Complication***	
Diabetic Nephropathy	80 (37.6%)
Diabetic Retinopathy	133 (62.4%

The frequency of undiagnosed hypertension in patients with diabetic micro vascular complications is shown in Table-II.

**Table-II:** Frequency of Undiagnosed Hypertension in Diabetic Patients with Microvascular Complications.

Undiagnosed Hypertension	Frequency (n)	Percent (%)
Yes	42	19.7%
No	171	80.3%

Total	213	100.0

The frequency of undiagnosed hypertension was significantly higher among obese patients with diabetic micro vascular complications as compared to non-obese patients (28.4% vs. 15.8%; p-value=0.032). However, there was no statistically significant difference in the frequency of undiagnosed hypertension across various subgroups based on patient’s age (p-value=0.750), gender (p-value=0.902), type of micro vascular complication (p-value=0.783) and duration of diabetes (p-value=0.763) as shown in Table-III.

**Table-III:** Stratification of Undiagnosed Hypertension across various Subgroups of Diabetic Patients with Microvascular Complications.

Characteristics	Participant (n=213)	Undiagnosed Hypertension n (%)	P-value
Age (years)	49.3±9.7		
>40 years	153 (71.8%)	31 (20.3%)	0.750
< 40 years	60 (28.2%)	11 (18.3%)	
***Gender***			
Male	125 (58.7%)	25 (20.0%	0.902
Female	88 (41.3%)	17 (19.3%)	
BMI (Kg/m2)	28.3±3.5		
Non-Obese	146 (68.5%)	23 (15.8%)	0.032*
Obese	67 (31.5%)	19 (28.4%)	
Duration of Diabetes (years)	8.2±3.9		
2-8 years	116 (54.5%)	22 (19.0%)	0.763
9-15 years	97 (45.5%)	20 (18%)	
***Micro vascular Complication***			
Diabetic Nephropathy	80 (37.6%)	15 (18.8%)	0.783
Diabetic Retinopathy	133 (62.4%	27 (20.3%)	

Chi-square test,

* observed difference was statistically significant.

## DISCUSSION

In the present study, undiagnosed hypertension was observed in 19.7% patients with diabetic micro vascular complications. The frequency of undiagnosed hypertension was significantly higher among obese as compared to non-obese patients (28.4% vs. 15.8%; p value=0.032. The results suggest that patients with microvascular complications can have a high incidence of hypertension which is silent but causing cardiovascular complications.

The management of diabetes mellitus (DM) requires addressing multiple goals – glycemic control and beyond.^10^ Major cause of mortality in diabetics is cardiovascular disease. Hypertension is one of the associated risk factors that multiplies the risk of CV disease in diabetics. Micro vascular complications occur as a result of exposure of vascular endothelium to high glucose levels leading to endothelial damage, oxidative stress due to superoxide overproduction, and the production of sorbitol and advanced glycation end-products due to hyperglycemia.^11^ These vascular changes in turn lead to decreased elasticity of vessel wall and narrowing of lumen leading to hypertension and cardiovascular disease.^11^-^13^ On the other hand, hypertension leads to increased development of complications and can further worsen it.^13^ Therefore it is not surprising that a recent study reported that a substantial proportion of diabetic patients with micro vascular complications had underlying undiagnosed hypertension and advocated routine screening of such patients in future practice.^8^ However, the available evidence was limited while there was no local published material which necessitated the present study. The objective of this study was to determine the frequency of undiagnosed hypertension among the diabetic patients with micro vascular complications.

In the present study, the mean BMI of diabetic patients was 28.3±3.5 Kg/m2 and 31.5% patients were obese. A study by Maheshwary N et al. reported similar mean BMI of 29.0±3.5 Kg/m2 among such patients at Combined Military Hospital, Lahore^14^ while Jain et al. reported it to be 28.3±2.2 Kg/m2 in Indian such patients.^15^ Our observation is also in line with that 2 other studies of Ali et al. and Ashraf et al. who reported similar frequency of 32.0% and 30.0% for obesity among such patients at Aga Khan University Hospital, Karachi and Ziauddin Medical University Hospital, Karachi respectively.^16^,^17^

In the present study, undiagnosed hypertension was observed in 19.7% patients with diabetic micro vascular complications. In a study conducted locally by Adnan M et al. frequency of previously undiagnosed hypertension was 25.3% which was in line with our findings.^18^ Our observation is also in line with that of a study conducted in 2010 by Rodrigues TC et al. who conducted a similar study in 188 Brazilian patients with diabetes associated micro vascular complications and found that 18.9% of the patients had undiagnosed hypertension.^8^ The incidence of hypertension increased with BMI; other factors like age, gender and type of micro vascular complication was not significantly related. In another study from Benghazi, hypertension was associated with older age, male sex, DM duration, and overweight in people with diabetes.^19^

The present study is a few of its kind in local population and adds to the limited already existing local and international research evidence on the topic. In the present study, a substantial proportion of patients with diabetic micro vascular complications had undiagnosed hypertension which is alarming and advocates routine blood pressure monitoring of such patients. This timely identification and anticipated management of underlying hypertension, which is not a routine practice in our population, will improve the outcome of such cases in future clinical practice. A new information which we observed was the presence of undiagnosed hypertension being significantly higher among obese patients which suggests cumulative effect of diabetes and obesity in the development of vascular changes and onset of hypertension. It endorses the use of patient’s BMI for risk stratification of such patients.

### Limitations:

It includes that we didn’t consider the effect of this timely diagnosis and management of hypertension on the long-term outcome of these patients which would have shed some light on the significance of this routine screening. Such a study is imperative and is highly recommended in future clinical research

## CONCLUSION

In the present study, a substantial proportion of patients with diabetic micro vascular complications suffered undiagnosed hypertension which is alarming and advocates routine blood pressure monitoring of such patients. It will ensure that that timely identification and anticipated management of underlying hypertension may improve the outcome of such cases in future clinical practice

### Author`s Contribution:

**SSQ:** Study design, planning, preparing the manuscript.

**WA:** Study design, writing results and discussion, supervision of study.

**MM:** Study design and data collection and analysis. Critical Review.

**MF:** Data collection, literature search, writing results, statistical analysis, manuscript writing.

**SKSK:** Concept, design, Data collection, results and statistics, manuscript writing.
